# Kentucky’s Proposed Reform of Her Penal Code

**Published:** 1910-03

**Authors:** 


### Abstracts and Selections.

### Kentucky's Proposed Reform of Her Penal Code.

All sociologists are agreed that reform not revenge should be the motive in the incarceration of criminals. There are some bills pending in the Kentucky legislature which have this humanitarian motive in view. One measure aims at separating confirmed criminals from those less advanced in evil-doing; another at reforming the vicious parole system, under which chicanery and favoritism are frequently instrumental in securing the release of hardened convicts at the expense of discipline, at the same time bringing the law into disrepute and removing from others the incentive to secure by meritorious behavior an early pardon. A more revolutionary measure still purposes to take from the jury the right of levying the penalty for cases of felony, investing the judge with discretionary power to fix the extent of punishment.

There is no doubt that our penal code needs revision. While much progress has been made during the past century when the horrors of English prisons first began to be realized, much still remains to be done. Vengeance has too long been the object of meting out so-called justice. The world is beginning to see that inhumanity to lawbreakers reflects most disastrously upon lawmakers, which, in the last analysis, are the people at large. Men permit the existence of vicious economic conditions and then punish those whom the conditions make into criminals. We need more men like Cooley, of Cleveland, and Lindsay, of Denver, to hasten the general recognition of man's inhumanity to man.—*Lancet Clinic.*

